# Cumulative Dis/Advantege, Structure, and Change: Three New Directions for CDA Research

**DOI:** 10.1093/geroni/igab046.1956

**Published:** 2021-12-17

**Authors:** William Dannefer, Chengming Hengming Han

**Affiliations:** Case Western Reserve University, Cleveland, Ohio, United States

## Abstract

Over the past several decades, evidence for cumulative dis/advantage as a regular feature of cohort aging has continued to cumulate, while new questions concerning the underlying dynamics continue to emerge. This paper reviews the accumulated knowledge base, and focused on three recently emerging lines of inquiry that hold great promise for expanding more fully our understanding of CDA processes: 1) the intersection of class stratification and race in the operation of CDA processes, 2) factors accounting for cross-national variations, and 3) the intersection of robust intracohort processed that generate cda with intercohort processes and the impact of historical and social change. These three new directions are briefly discussed.

